# Self-labelling enzymes as universal tags for fluorescence microscopy, super-resolution microscopy and electron microscopy

**DOI:** 10.1038/srep17740

**Published:** 2015-12-08

**Authors:** Viktoria Liss, Britta Barlag, Monika Nietschke, Michael Hensel

**Affiliations:** 1 Abt. Mikrobiologie, Universität Osnabrück, Osnabrück, Germany

## Abstract

Research in cell biology demands advanced microscopy techniques such as confocal fluorescence microscopy (FM), super-resolution microscopy (SRM) and transmission electron microscopy (TEM). Correlative light and electron microscopy (CLEM) is an approach to combine data on the dynamics of proteins or protein complexes in living cells with the ultrastructural details in the low nanometre scale. To correlate both data sets, markers functional in FM, SRM and TEM are required. Genetically encoded markers such as fluorescent proteins or self-labelling enzyme tags allow observations in living cells. Various genetically encoded tags are available for FM and SRM, but only few tags are suitable for CLEM. Here, we describe the red fluorescent dye tetramethylrhodamine (TMR) as a multimodal marker for CLEM. TMR is used as fluorochrome coupled to ligands of genetically encoded self-labelling enzyme tags HaloTag, SNAP-tag and CLIP-tag in FM and SRM. We demonstrate that TMR can additionally photooxidize diaminobenzidine (DAB) to an osmiophilic polymer visible on TEM sections, thus being a marker suitable for FM, SRM and TEM. We evaluated various organelle markers with enzymatic tags in mammalian cells labelled with TMR-coupled ligands and demonstrate the use as efficient and versatile DAB photooxidizer for CLEM approaches.

In biological research, especially in cell biology, determination of the precise subcellular localization of a given protein is of central importance for understanding its function. In many cases confocal fluorescence microscopy (FM) of fluorophore fusions is not sufficient to precisely clarify the localization of a protein. The optical resolution limit of conventional light microscopy restricts the localization precision to 200 nm in x and y direction. While technical developments in light microscopy and the introduction of super-resolution microscopy (SRM) enhances the resolution limit up to 20 nm^1^,^2^, SRM and all other FM techniques fail in providing information on the subcellular context of a protein. In such cases, transmission electron microscopy (TEM) is the best way to locate a protein with a resolution of 1 nm and within its subcellular context^3^. However, EM markers are needed for the identification of a protein of interest on TEM sections, which up to now is still challenging. On the other hand, the implementation of EM alone to study the biological role of a protein is not sufficient, since only live cell imaging can reveal the temporal and spatial dynamics of a protein within a comparatively big sample. For these two reasons TEM is often combined with live cell FM or also SRM, meaning live cell correlative light and electron microscopy (live cell CLEM), to correlate the ultrastructure with the fluorescence signal of a protein of interest for identification and to perform live cell analyses before EM^4^,^5^,^6^,^7^,^8^,^9^,^10^. Nevertheless in a correlative approach with TEM the resolution of the fluorescence signal of a fusion protein is always the limiting factor for a precise localization of the protein, with SRM having an advantage over FM. To overcome this drawback up to now only few genetically encoded markers were characterized, which in addition to light microscopic techniques are applicable as EM markers. All of these CLEM markers take advantage of the well-established diaminobenzidine (DAB) oxidation. Therefore DAB is locally oxidized either during an enzymatic reduction of H_2_O_2_ by peroxidases or by singlet oxygen (^1^O_2_) produced during the illumination of specific fluorescent labels (photooxidation), both leading to the generation of an osmiophilic DAB polymer, which is detectable on TEM sections after staining with osmium (OsO_4_)^9^,^10^,^11^,^12^,^13^,^14^. Unfortunately, most of the known genetically encoded live cell-CLEM markers based on DAB oxidation have limitations, and to our knowledge only three general-purpose-markers for correlative approaches of all microscopic techniques were described (Fig. 1), i) ReAsH^15^,^16^,^17^, ii) resorufin ligase^18^ and iii) FLIPPER^19^. However for those three markers some constraints were reported, such as nonspecific labelling and cellular toxicity for ReAsH^15^,^16^,^17^, efficiency problems for the resorufin ligase^18^ and restricted functionality for FLIPPER^19^.

In this study we introduce new general-purpose-markers for correlative live cell approaches. We provide results which demonstrate their multimodal use for FM, SRM and EM. The widely applicable HaloTag^20^ (Promega), SNAP-tag^21^ and CLIP-tag^22^ (New England Biolabs) systems represent self-labelling enzyme tags which catalyse the covalent attachment of an exogenously added synthetic ligand. Such synthetic ligands are tag specific and can be coupled to diverse useful labels, such as fluorescent dyes, affinity handles, or solid surfaces. The covalent attachment of the functionalized ligand to the enzyme tag is highly specific, happens rapidly under physiological conditions in living cells, or in chemically fixed cells, and is most importantly irreversible. HaloTag is a haloalkane dehalogenase that reacts irreversibly with primary alkylhalides. SNAP-tag, an *O*^*6*^-alkylguanine-DNA-alkyltransferase, reacts with *O*^*6*^-benzylguanine derivatives, while CLIP-tag, an *O*^*2*^-alkylcytosine-DNA-alkyltransferase, reacts with *O*^*2*^-benzylcytosine derivatives. In addition, the genetically encoded tags can be expressed in prokaryotic and eukaryotic cells^20^,^21^,^22^,^23^,^24^,^25^. One of the ligand bound labels is the red fluorescent rhodamine derivate TMR (tetramethylrhodamine) (ex. 545, em. 575). TMR is not toxic to cells, membrane-permeable, monomeric and can be used for FM and SRM^20^,^26^. Additionally, Perkovic *et al.*^26^ demonstrated that the TMR ligand preserves its fluorescence during the high-pressure freezing/freeze substitution (HPF/FS) EM-preparation protocol independently of the uranyl acetate concentration, thus allowing post-embedding correlation of FM and SRM with both TEM and scanning EM (SEM) samples.

Here, we show that the highly specific TMR ligands of HaloTag, SNAP-tag or CLIP-tag can, in addition to the fluorescent properties, photooxidize DAB to an osmiophilic polymer visible on TEM sections, thus displaying all-in-one markers suitable for all three microscopic modalities (Fig. 1). The DAB photooxidation by rhodamine was first reported by Sandell and Masland^27^ for immuno-labelled cultured cells, and was later described as successful also for other rhodamine derivatives^27^,^28^,^29^,^30^. We evaluated the TMR ligands for HaloTag, SNAP-tag and CLIP-tag fused to several organelle markers in mammalian cells and provide for the first time results for its use as DAB photooxidizer for EM.

## Results

### TMR ligands as multimodal microscopy marker

**Figure 1:**
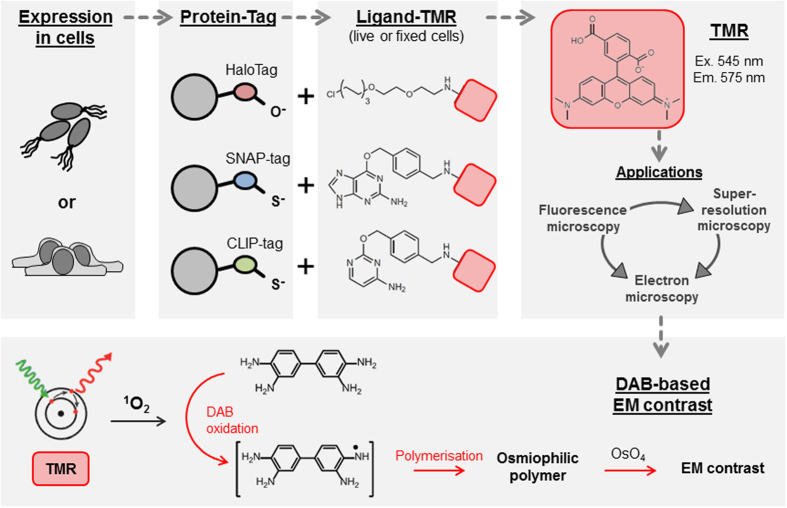
TMR ligands for genetically encoded tags HaloTag, SNAP-tag and CLIP-tag and applications in microscopy. Proteins of interest are tagged with genetically encoded markers HaloTag (Promega), SNAP-tag (NEB) or CLIP-tag (NEB) and expressed in prokaryotic or eukaryotic cells. Specific ligands for each tag consist of a ligand conjugated to tetramethylrhodamine (TMR) that are added to living or fixed cells to covalently label the tagged protein of interest. After labelling with TMR various analyses are possible, such as fluorescence microscopy, super-resolution microscopy (SRM) or electron microscopy (EM), with the option to perform correlative light and electron microscopy (CLEM). For EM, diaminobenzidine (DAB) is locally oxidized by singlet oxygen (^1^O_2_) produced during the illumination of TMR. The resulting osmiophilic polymer subsequently is stained with osmium (OsO_4_).

The fluorochrome rhodamine has been used to mediate photooxidation of DAB. Tetramethylrhodamine (TMR) is a derivative of rhodamine. TMR-labelled ligands are available for the three self-labelling enzyme tags HaloTag, SNAP-tag and CLIP-tag. We considered that TMR might also be an attractive label for CLEM. After the reaction of genetically tagged proteins of interest with TMR-conjugated ligands, the fusion protein can be detected by fluorescence microscopy techniques such as epifluorescence microscopy or super-resolution microscopy (SRM). The cell-permeable properties of TMR ligands will allow live cell imaging experiments. By virtue of the TMR-mediated DAB photooxidation, the formation of DAB polymers should also allow the localization of the fusion protein by ultrastructural approaches. Thus, use of these self-labelling enzymes as genetically encoded tags and TMR-conjugated ligands could provide a novel multimodal toolbox for CLEM (see Fig. 1 for schematic representation).

For the evaluation of the toolbox, we generated several constructs for the expression of eukaryotic organelle markers. LAMP1 and complex V γ subunit fusion proteins were used for localization at late endosomes and inner mitochondrial membrane, respectively. Fusion to the LifeAct peptide allowed to label the F-actin cytoskeleton. Further constructs contained targeting sequences for localization at the Golgi apparatus or endoplasmic reticulum (ER), or a palmitoylation site for localization at the plasma membrane. The organelle markers were fused to HaloTag, and in some cases additionally to meGFP for comparison of localization patterns of meGFP and the TMR ligand. In addition, several organelle markers were fused to SNAP-tag or CLIP-tag (overview in Fig. S1A). We initially tested all constructs for expression, localization and labelling specificity in HeLa cells. For FM labelling was performed in living cells for 15 min. at 37 °C in cell culture medium with a TMR ligand concentration of 100 nM. As shown in Fig. S1BCD, all organelle marker fusions tested revealed an organelle-specific localization without background staining.

We further tested some of the constructs for functionality in SRM by dSTORM (direct stochastic optical reconstruction microscopy) (Fig. S2). Labelling with the TMR ligand was performed with a reduced concentration of 20 nM. All markers showed expected localization with much higher resolution compared to the diffraction-limited images.

**Figure 2:**
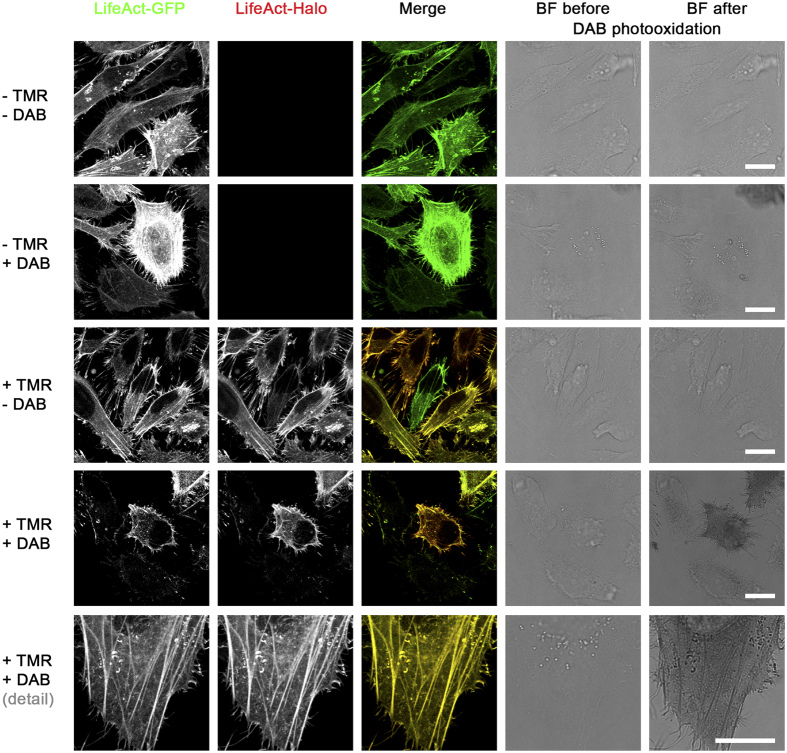
DAB photooxidation by TMR-labelled HaloTag fusion protein. HeLa cells stably transfected with pLifeAct-HaloTag-meGFP were incubated with medium only (−TMR) or medium containing 100 nM ligand HTL-TMR (+TMR) for 15 min. to label the F-actin cytoskeleton. After fixation and quenching cells were covered with buffer only (−DAB) or fresh DAB solution in buffer (+DAB). During illumination of samples on the microscope stage, cells labelled with HTL-TMR and incubated with DAB showed the formation of localized DAB polymers after 8–12 min. concomitantly with disappearance of fluorescence signals. Notice the precise staining of actin stress fibres by DAB polymers in details image (bottom). Scale bars: 20 μm.

For application in ultrastructural analyses, a genetically encoded marker is required to generate electron density. We found that TMR, as other fluorochromes, can be used in photooxidation of DAB to an osmiophilic polymer. DAB tetrahydrochloride is a water-soluble and membrane-permeable monomer. Photooxidation of DAB occurs by oxidation by singlet oxygen (^1^O_2_) that is generated during illumination of fluorochromes. This reaction leads to a brown, insoluble and granular DAB polymer which is membrane impermeable^11^,^13^. This DAB polymer is visible in transmitted light microscopy (TLM) and on TEM sections after a staining with electron dense osmium (OsO_4_) (Fig. 1). We evaluated DAB photooxidation by TMR ligands first by TLM for HeLa cells stably expressing LifeAct-HaloTag-meGFP. At the beginning we tested various concentrations of HaloTag ligand coupled to TMR ranging from 0.1 nM to 1,000 nM (Fig. S3). We could show that a convincing DAB photooxidation by TMR occurs already with 10 nM TMR ligand. Thereby a sufficient amount of DAB precipitates was formed after 12 min. with 10 nM TMR ligand and after 8–10 min. with 100 or 1,000 nM TMR ligand, with no visible difference between 100 nM and 1,000 nM. Since the fluorescence signal of 10 nM TMR was too weak for light microscopy, we decided to proceed with 100 nM for further experiments. Next we investigated HeLa cells expressing various organelle markers fused to self-labelling enzyme tags. An example of HeLa cells stably expressing LifeAct-HaloTag-meGFP is shown in Fig. 2. During illumination of the samples using green light, samples labelled with TMR ligand and incubated with DAB showed formation of localized DAB polymers after 8–12 min. (Fig. S4B). DAB polymer formation was depending on the density of the organelle marker and occurs in parallel to decay of TMR fluorescence. Samples without TMR labelling or without incubation with DAB solution showed no DAB polymerization. DAB photooxidation by GFP was previously reported^31^, however under the conditions applied in our study, the meGFP tag alone was not sufficient to photooxidize DAB (Fig. 2). Various fusion proteins with other organelle markers with or without an additional meGFP tag were tested for DAB photooxidation and showed comparable results (Fig. S4). In order to extend the toolbox of self-labelling enzymes for DAB photooxidation, we generated various fusion proteins with SNAP-tag or CLIP-tag. We observed that labelling of SNAP-tag or CLIP-tag with ligands BG-TMR or BC-TMR, respectively, allowed detection by fluorescence microscopy as well as DAB photooxidation (Fig. S5BC). In addition to TMR we tested further fluorescent ligands for DAB photooxidation, i.e. Dy547 (ex. 554 nm, em. 568 nm), SiR (ex. 650 nm, em. 668 nm) and Atto655 (ex. 663 nm, em. 684 nm). We observed that Dy547 was also able to induce photooxidation that was delayed compared to TMR, while SiR and Atto655 were not able to photooxidize DAB within the time frame used (Fig. S5).

### Correlative Light and Electron Microscopy with TMR ligands

**Figure 3:**
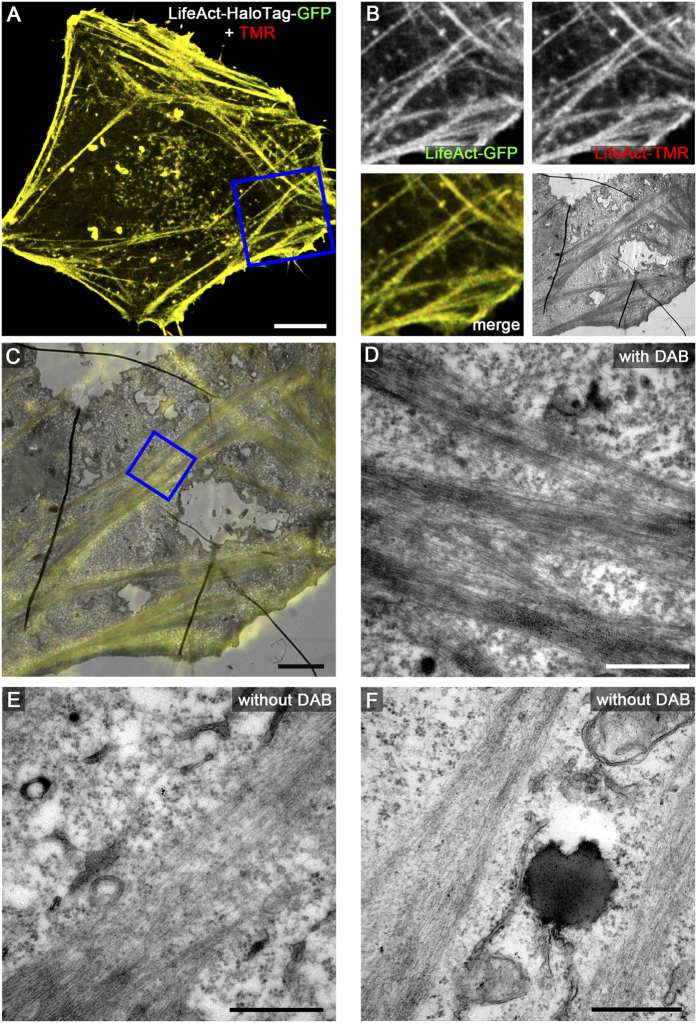
HaloTag-TMR as genetically encoded CLEM marker for actin filaments. HeLa stably expressing LifeAct-HaloTag-meGFP were seeded on a Petri dish with a gridded coverslip. Next day cells were incubated with 100 nM HTL-TMR for 15 min. to label HaloTag followed by live cell imaging using CLSM. The cell of interest ((**A**) MIP) was imaged and immediately fixed on the microscope stage. DAB photooxidation by TMR was performed and samples were prepared for TEM. Details of LifeAct-HaloTag-meGFP and TMR-positive actin stress fibres are shown by correlative live cell CLSM ((**B**) single Z plane) and TEM (**C**,**D**) micrographs. (**D**) Higher magnification of actin stress fibres with DAB incubation. (**E**,**F**) show control cells with higher magnification of actin stress fibres without DAB incubation. Actin stained with DAB appears more electron-dense. Scale bars: 10 μm (**A**), 2 μm (**B**,**C**), 500 nm (**D**–**F**).

**Figure 4:**
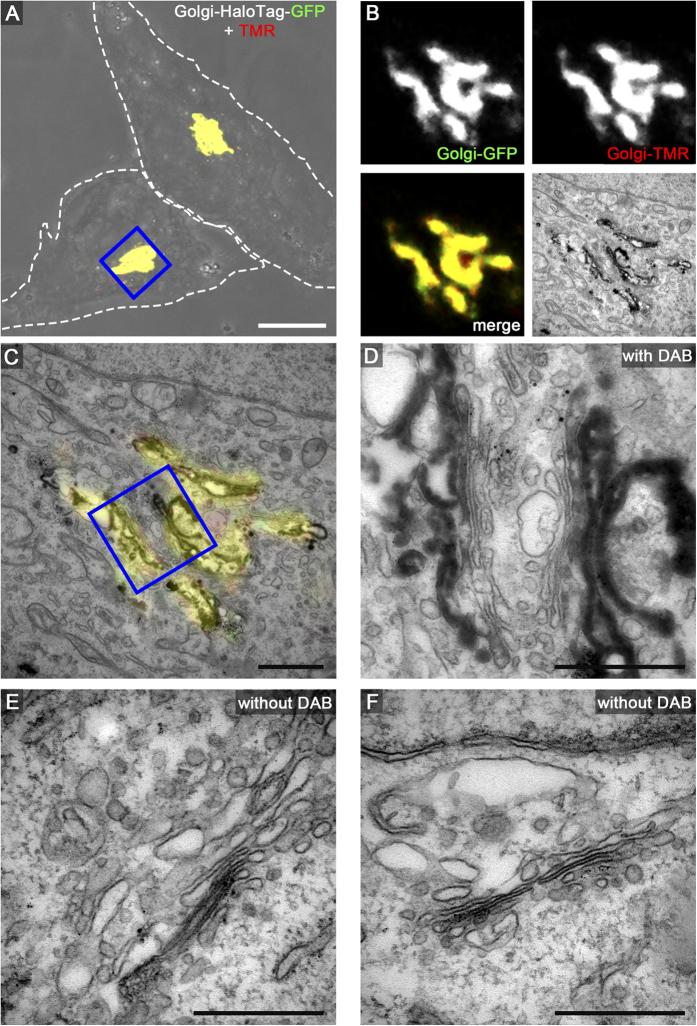
HaloTag-TMR as genetically encoded CLEM marker for proteins in the Golgi. HeLa cells transiently transfected with Golgi-HaloTag-meGFP were seeded on a Petri dish with a gridded coverslip and treated as described for Fig. 3. Scale bars: 10 μm (**A**), 1 μm (**B**,**C**), 500 nm (**D**–**F**).

**Figure 5:**
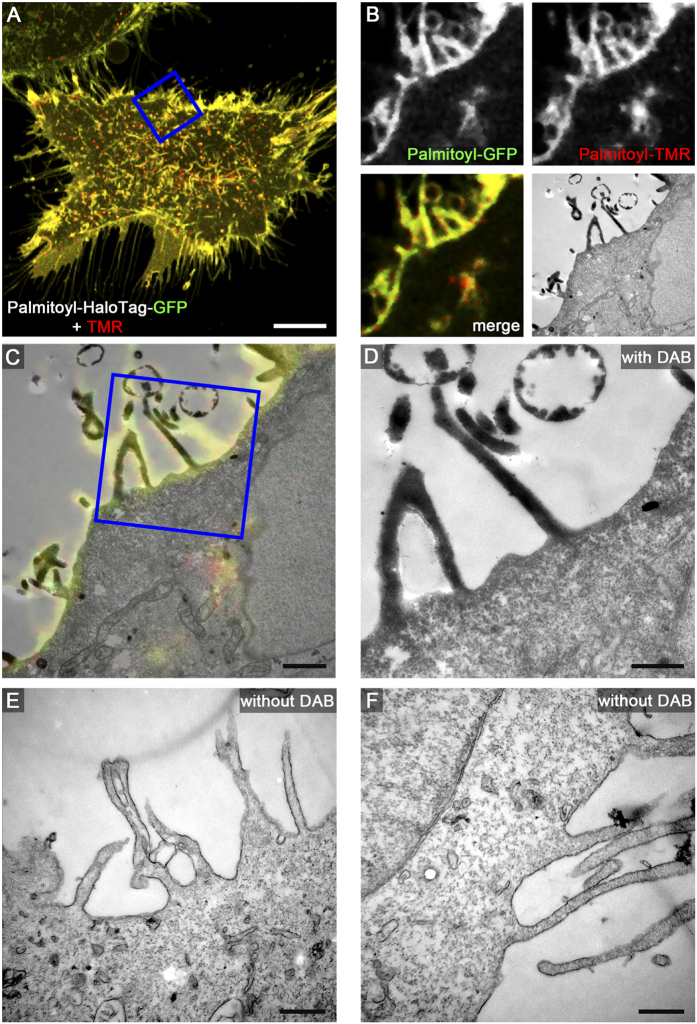
HaloTag-TMR as genetically encoded CLEM marker for proteins at the plasma membrane. HeLa cells transiently transfected with Palmitoyl-HaloTag-meGFP were seeded on a Petri dish with a gridded coverslip and treated a as described for Fig. 3. Scale bars: 10 μm (**A**), 1 μm (**B**,**C**), 500 nm (**D**–**F**).

**Figure 6:**
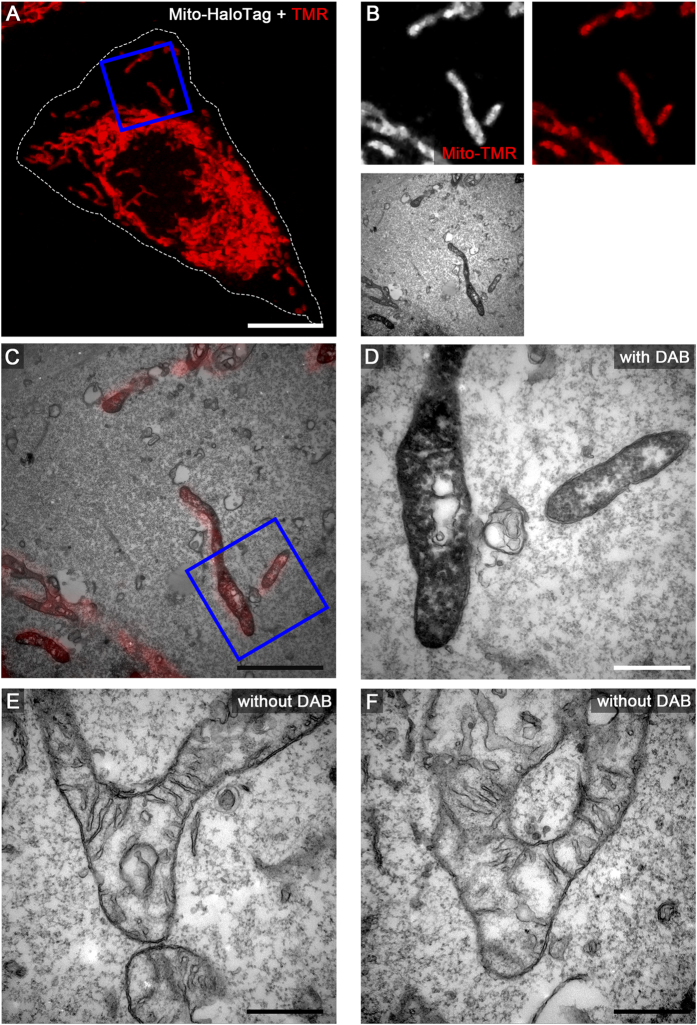
HaloTag-TMR as genetically encoded CLEM marker for mitochondrial proteins. HeLa cells transiently transfected with Mito-HaloTag (complex V tagged at mitochondrial matrix site) were seeded on a Petri dish with a gridded coverslip and treated as described for Fig. 3. Scale bars: 10 μm (**A**), 2 μm (**B**,**C**), 500 nm (**D**–**F**).

We next applied several of the organelle markers fused to HaloTag to CLEM analysis using live cell fluorescence imaging and TEM of ultrathin sections. This includes HeLa cells expressing LifeAct-HaloTag-meGFP (Fig. 3), Golgi-HaloTag-meGFP (Fig. 4), Palmitoyl-HaloTag-meGFP (Fig. 5), Mito-HaloTag (Fig. 6), and LAMP1-HaloTag-meGFP (Fig. S6). Cells were incubated with 100 nM HTL-TMR for 15 min. to label the HaloTag followed by live cell imaging using CLSM. Next rapid fixation was performed on the microscope stage. After DAB photooxidation by TMR for 8–12 min. the samples were prepared for TEM. Control cells without DAB photooxidation demonstrate the difference between stained and unstained structures of interest. As visible on ultrathin TEM sections, each of the CLEM organelle marker tested showed strong formation of DAB polymers, resulting in an electron-dense OsO_4_ signal corresponding to the HTL-TMR fluorescence signal of the marker. The examples shown in Fig. 3 to Fig. 6 and Fig. S6 clearly demonstrate that fluorescence signals of HaloTag fusion proteins in live cell imaging can be precisely correlated with ultrastructural information obtained by TEM of ultrathin sections. DAB photooxidation was also possible with TMR-conjugated ligands of SNAP-tag and CLIP-tag and for CLEM experiments we observed performance of these tags similar to HaloTag (data not shown).

A critical parameter of DAB photooxidation is the adjustment of the amount of DAB polymer. If periods of photooxidation were too long, the massive accumulation of OsO_4_ prevented proper localization of structures of interest. Since DAB polymer formation is a function of the expression level of the tagged protein, the light intensity and the duration of illumination, as well as DAB concentration, oxygen level of DAB solution and the OsO_4_ contrasting, optimal parameters for photooxidation have to be established empirically. It was observed that DAB polymers slightly diffuse from the polymerization site, resulting in loss of signal intensity and reduced localization precision for the labelled protein of interest. Tagged marker proteins with cytosolic localization resulted in less precise DAB staining due to diffusion. For example, Palmitoyl-HaloTag-meGFP was detected on the cytosolic face of the entire plasma membrane by fluorescence microscopy, but DAB polymers were most pronounced at regions of high local density of this marker, i.e. in the lumen of filopodia (Fig. 5). We found that F-actin stress fibres are very dense packed protein structures causing strong DAB polymerization after labelling with LifeAct-HaloTag-meGFP. Marker proteins with tags exposed to the lumen of a membrane compartment provided very intense formation of DAB polymers. Golgi-HaloTag-meGFP (Fig. 4) or LAMP1-HaloTag-meGFP (Fig. S6) generated strong luminal signals, most likely due to the highly reduced diffusion of the DAB polymers formed.

Taken together, the results shown here demonstrate the simple and convenient use of TMR ligands of self-labelling enzyme tags as DAB-photooxidizing CLEM marker.

## Discussion

The red fluorescent dye TMR can be used as a fluorochrome bound to ligands of the widely applicable HaloTag, SNAP-tag and CLIP-tag and these systems are already well established as reliable and versatile tags for FM and SRM^20^,^21^,^22^,^23^. In this study we introduced the TMR-conjugated ligands as DAB-based EM markers, thus extending the use of HaloTag, SNAP-tag and CLIP-tag as general-purpose markers suitable for correlative microscopy of all three microscopic techniques. Up to now, only some genetically encoded CLEM markers based on DAB oxidation exist and, to our knowledge, only three of them were reported to work as general-purpose-marker.

Unfortunately, most of the previously reported DAB-based live cell CLEM markers have certain limitations (Table 1) and not all DAB-based CLEM markers are applicable for SRM. The most important disadvantage of HRP (horseradish peroxidase) and APEX/APEX2 (enhanced ascorbate peroxidase) is the lack of intrinsic fluorescence. This can be compensated by addition of a further fluorescent protein or tag. A similar approach was done for FLIPPER (fluorescent indicator and peroxidase for precipitation with EM resolution), which consists of HRP fused to different fluorophores, such as EGFP, mTurquoise2, mOrange2 or mCherry^19^. Multiple tags are used to combine various modalities or to increase signal intensities, but this strategy results in very large CLEM markers, that can lead to a false protein localization. We observed that single enzyme tags after TMR labelling provide sufficient signal intensities for FM as well as rapid DAB photooxidation, negating the need for multiple tags. One further problem of HRP and HRP-based FLIPPER is the restriction of their functionality. Both are not functional in the cytosol due to the reducing environment^19^,^32^,^33^,^34^,^35^. Although APEX/APEX2 is a very robust enzyme and functional in all cellular compartments, it requires heme for its function resulting in potential limitations^36^,^37^. One further marker reported for CLEM, the resorufin ligase, which catalysed the site-specific and covalent attachment of the red dye resorufin to a genetically encoded 13-aa recognition peptide, requires ATP for its function in addition to a co-expression of both peptide and ligase^18^. In contrast, self-labelling enzymatic tags such as HaloTag, SNAP-tag and CLIP-tag are functional in all cellular compartments and are independent of co-factors like heme (APEX/APEX2), or ATP, or the expression of an additional enzyme (resorufin ligase). By virtue of the various ligands, self-labelling tags are applicable not only for a range of imaging techniques, but also for protein pull-down assays, protein detection in SDS-PAGE, flow cytometry, or high throughput binding assays in microtiter plates. In addition, compared to other known subcellular markers, enzymatic tags have the great advantage to be strictly monomeric^38^. In contrast to fluorescent proteins such as GFP or miniSOG there is no maturation required for fluorochromes like TMR, thus allowing analyses of highly dynamic processes. Furthermore, TMR shows a strong fluorescence and a negligible bleaching rate, whereas miniSOG possess only weak and fast bleaching fluorescence^39^,^40^,^41^. Also resorufin was stated not to be highly optimal for long-term fluorescence imaging^18^. Another advantage of the TMR labelling system is that it is not toxic to cells^20^,^26^, whereas the 4Cys-ReAsH labelling system requires antidotes due to arsenic toxicity, in addition to its non-covalent and unspecific labelling^15^,^16^,^17^.

For EM, TMR is able to photooxidize DAB to an osmiophilic polymer which can be made electron-dense by staining with OsO_4_. We tested several eukaryotic organelle markers fused to HaloTag, SNAP-tag or CLIP-tag and labelled with TMR ligands for detection by FM, SRM and TEM, and additionally correlative FM and TEM. The live cell-CLEM results demonstrate that each organelle marker tested showed a strong electron dense DAB polymerization signal corresponding to the TMR fluorescence signal of the marker protein. Some of the tested constructs additionally contained meGFP for the comparison of labelling specificity, but we could clearly exclude that GFP was responsible for the DAB photooxidation as reported^31^. Fusion proteins without an additional meGFP showed after TMR labelling a similar DAB photooxidation (Fig. S4). We also tested several other fluorochromes conjugated to the HaloTag ligand, but none of these achieved photooxidation as efficient as TMR (Fig. S5)

In general, DAB polymers can slightly diffuse from the polymerization site, thereby reducing the localization precision of the marker. Peroxidase-based DAB-oxidizer lead to a higher polymer diffusion compared to light-dependent DAB photooxidizer, whereas enzymatic marker are more sensitive^9^. We found that TMR photooxidizes DAB to locally visible polymers within 8–12 min. Highly abundant and densely packed proteins like actin within actin stress fibres displayed stronger DAB polymerization without obvious polymer diffusion. In case of proteins with cytosolic localization, the diffusion of DAB polymer is more pronounced. However, this is a general problem affecting all CLEM markers relying on DAB polymerization. Optimization has to be done for individual proteins to reduce the diffusion of DAB polymers. This may be achieved by strong chemical crosslinking prior to the formation of the DAB polymer, low temperatures during the DAB photooxidation, and increased levels of exogenously added oxygen^14^. DAB photooxidation by proteins localized within the lumen of organelles like Golgi or mitochondria is clearly delimited due to the adjacent membranes. One limitation of all light-dependent DAB-based markers is the restriction for small and thin samples, due to the confined light available in the microscopy, whereas enzyme-dependent DAB-based markers can also work for thick tissues.

In summary, the TMR labelling system showed a fast and sensitive labelling in applications under physiological conditions. The TMR fluorescence is strong and the DAB photooxidation by the TMR ligand is fast and provides a convincing staining for EM. In addition, it was shown that TMR preserves its fluorescence during the HPF/FS EM preparation protocol, thus allowing post-embedding correlation of FM and SRM with both TEM and SEM samples^26^. So far, TMR-conjugated ligands and the genetically encoded tags HaloTag, SNAP-tag, or CLIP-tag probably represent the easiest to use and most versatile labelling system for live cell-CLEM.

## Materials and Methods

### Cell lines and cell culture

The non-polarized epithelial cell line HeLa (American Type Culture Collection, ATCC no. CCL-2) was cultured in Dulbecco’s modified Eagle’s medium (DMEM) containing 4.5 g × l^−1^ glucose, 4 mM stable glutamine and sodium pyruvate (Biochrom) and supplemented with 10% inactivated fetal calf serum (iFCS) (Sigma-Aldrich) at 37 °C in an atmosphere containing 5% CO_2_ and 90% humidity. The stably transfected HeLa cell line expressing LifeAct-HaloTag-meGFP was cultured under same conditions. The transfection vectors with various combinations of organelle markers and tags are listed in Table S2. The generation of the plasmids is described in detail in Suppl. Material and oligonucleotides and synthetic DNA used for the construction are listed in Table S3.

### HeLa cells transient transfection

HeLa cells were cultured for one day and transfected with FUGENE HD reagent (Promega) according to manufacturer’s instruction. In brief, 0.5–2 μg of plasmid DNA were solved in 25–100 μl DMEM without iFCS and mixed with 1–4 μl FUGENE reagent (ratio of 1:2 for DNA to FUGENE). After 10 min. incubation at room temperature (RT) the transfection mix was added to the cells in DMEM with 10% iFCS for at least 18 h. Then the cells were provided with fresh medium without transfection mix.

### Labelling of enzymatic tags with TMR ligand

The self-labelling of the organelle markers fused to HaloTag, SNAP-tag or CLIP-tag enzymes was performed in living cells at 37 °C 24 h after transfection of the cells by adding the appropriate ligand to the cell culture medium for 15 min. Beside the main HaloTag-ligand TMR (ex. 545 nm, em. 575 nm) (Promega) we also investigated HaloTag-ligands with Dy547 (ex. 554, em. 568) (Div. Biophysics, University Osnabrück), SiR (ex. 650 nm, em. 668 nm)^42^ or Atto655 (ex. 663 nm, em. 684 nm)^25^. For labelling with the non-membrane permeable Dy547 ligand, cells had to be fixed (3% PFA) and permeabilized (0.1% saponin in PBS). The concentration of ligands was ranging from 0.1 nM to 1 μM and concentrations of 20 nM for SRM and 100 nM for FM and EM were found most suitable after evaluation. After labelling the cells were washed trice with warm PBS and prepared for live cell FM or SRM.

### Live cell imaging

For live cell imaging DMEM was replaced by imaging-medium consisting of Minimal Essential Medium (MEM) with Earle’s salts, without NaHCO_3_, without L-glutamine and without phenol red (Biochrom) supplemented with 30 mM HEPES (4-(2-hydroxyethyl)-1-piperazineethanesulfonic acid) (Sigma-Aldrich), pH 7.4. The imaging studies were mainly performed using the confocal laser-scanning microscope (CLSM) Leica SP5 equipped with an incubation chamber maintaining 37 °C and humidity during live cell imaging and several objectives, such as 10× (HC PL FL 10×, NA 0.3), 20× (HC PL APO CS 20×, NA 0.7), 40× (HCX PL APO CS 40×, NA 1.25–0.75) and 100× objective (HCX PL APO CS 100×, NA 1.4–0.7) and the polychroic mirror TD 488/543/633 for the three channels GFP/ RFP/ BF (Leica, Wetzlar, Germany). The illumination of samples at the Leica SP5 for DAB photooxidation of fixed cells occurred with an OSRAM HXP-R120W/45C VIS lamp (used power 70 mW) with green light (filter excitation wavelength 515–560 nm). The software LAS AF (Leica, Wetzlar, Germany) was used for setting adjustment, image acquisition and image processing. Due to requirements for matching filters, one experiment was performed at a Zeiss Cell Observer Spinning Disk microscope (SDM) equipped with a Yokogawa Spinning Disc Unit CSU-X1a 5000 and an Evolve EMCCD camera (Photometrics, USA). A 63× objective (alpha Plan-Apochromat 63×, NA 1.46) was used. Images were acquired using the ZEN software (Zeiss) and the following filter combinations: GFP with BP 525/50, RFP with HC 593/46 and FRFP with BP 690/50. All images obtained were processed by ZEN software. The illumination of samples at the Zeiss SDM for DAB photooxidation of fixed cells occurred with an OSRAM HXP-R120W/45C VIS lamp (used power 50 mW) with either green light (filter excitation wavelength 537–563 nm) or red light (filter excitation wave length 625–655 nm).

### Super-resolution microscopy

TIRF microscopy was performed with an inverted Olympus IX71 microscope equipped with a motorized quad-line total internal reflection (TIR) illumination condenser (Olympus), with 488 nm (250 mW), 561 nm (150 mW) and 647 nm (250 mW) lasers (Olympus) as well as a back-illuminated EM-CCD camera (Andor iXon Ultra 897). A 150× objective plus 1.6× magnification with a numerical aperture of 1.45 (UAPON 150×/1.45, Olympus) was used for TIR-illumination. The excitation beam was reflected into the objective by a quad-line dichroic beam splitter for reflection at 405 nm, 488 nm, 568 nm and 647 nm (Di01 R405/488/561/647, Semrock). 1,000 frames were recorded with an exposure time of 32 ms for 561 nm and laser power of 33 mW. For oxygen depletion 100 mM β-mercaptoethylamine, 4.5 mg × ml^−1^ D glucose, 0.04 mg × ml^−1^ catalase and 0.5 mg × ml^−1^ glucose-oxidase were added in 1 ml PBS. Localization of single molecules as well as single molecule tracking were carried out as previously described^25^,^43^,^44^.

### Sample preparation for CLEM and DAB photooxidation

HeLa cells (1 × 10^5^) were seeded in a Petri dish with a gridded coverslip (MatTek, Ashland, MA) two days prior to microscopy. On the second day cells were transfected, if necessary. On the third day cells were observed by live cell imaging and fixed as fast as possible directly on stage with pre-warmed 2.5% glutaraldehyde (Electron Microscopy Sciences) in buffer (0.2 M HEPES, pH 7.4, 5 mM CaCl_2_) for 1 h at 37 °C. After fixation cells were rinsed several times in buffer containing 50 mM glycine and 20 mM potassium cyanide, to reduce unspecific DAB staining, followed by rinses in buffer. For the DAB photooxidation fixed cells expressing HaloTag, SNAP-tag or CLIP-tag fusion proteins were covered with freshly-prepared ice-cold 1 mg × ml^−1^ DAB in 0.2 M HEPES buffer and the sample was viewed again by CLSM. DAB photooxidation was started by illumination the ROI with green light (Xenon lamp, full power) until a brown DAB polymer was visible by eye, mostly 8–12 min. After DAB oxidation the DAB solution was removed and the cells were washed several times in HEPES buffer. Post-fixation was performed with 2% osmium tetroxide (Electron Microscopy Sciences) in buffer containing 1.5% potassium ferricyanide (Sigma) and 0.1% ruthenium red (Applichem) for 1 h at 4 °C in the dark. After several washing steps the cells were dehydrated in a cold graded ethanol series and finally one rinse in anhydrous ethanol and two rinses in anhydrous acetone at room temperature. The gridded coverslip was removed from the Petri dish and cells were flat-embedded in EPON812 (Serva). During the removal of the gridded coverslip from the polymerized EPON block the engraved coordinates were transferred to the EPON surface and allowed trimming around the ROI. Serial 70 nm sections were cut with an ultramicrotome (Leica EM UC6) and collected on formvar-coated EM copper grids. After staining with uranyl acetate and lead citrate, cells were observed with TEM (Zeiss EFTEM 902 A), operated at 80 kV and equipped with a 2 K wide-angle slow-scan CCD camera (TRS, Moorenwies, Germany). Images were taken with the software ImageSP (TRS image SysProg, Moorenwies, Germany). For image analysis, software packages LAS AF (Leica. Wetzlar), ImageJ (http://rsbweb.nih.gov/ij/) and Imaris (Bitplane, Zürich) were used. Stitching and overlay of CLSM and TEM images was done using Photoshop 5.5 (Adobe) according to Keene *et al.*^45^. Briefly, a new file (RGB colour, 8 bit, 300 dpi) was opened in Photoshop and the TEM micrograph was pasted into this new file. In a second step the fluorescence image was pasted into as file as another top layer. The opacity of the fluorescence image was adjusted to approximately 30–35% so that the TEM image on the bottom layer became visible. Next, the fluorescence image is aligned in size and rotation to the TEM image and in some cases adjustments in contrast and brightness were performed. Finally both layers are cropped to a desired size.

## Additional Information

**How to cite this article**: Liss, V. *et al.* Self-labelling enzymes as universal tags for fluorescence microscopy, super-resolution microscopy and electron microscopy. *Sci. Rep.* **5**, 17740; doi: 10.1038/srep17740 (2015).

## Supplementary Material

Supplementary Information

## Figures and Tables

**Table 1:** Genetically encoded markers based on DAB oxidation and application in EM, FM and SRM.

Marker (MW)	EM	FM	SRM	Limitations	Reference
HRP (36 kDa)	P*	−**	−	Not fluorescent. Not functional in cytosol.	32-35
APEX/APEX2 (28 kDa)	P	−	−	Not fluorescent. Heme-dependent.	36,37
GFP (26.9 kDa)	L	+	−	DAB photooxidation was described as negligible.	31,46,47
				DAB photooxidation restricted for small samples.	
miniSOG (15.4 kDa)	L	+	−	Very weak fluorescence and rapid bleaching.	39-41,48
				DAB photooxidation restricted for small samples.	
4Cys + ReAsH (Recognition peptide: 6–9 aa)	L	+	+	Due to toxicity arsenic antidotes required. Non-covalent, unspecific labelling of ligand.	15-17
				DAB photooxidation restricted for small samples.	
Resorufin ligase (Recognition peptide: 13 aa)	L	+	+	ATP-dependent. Simultaneous co-expression of peptide and ligase. Resorufin not optimal for long-term fluorescence imaging or for highly efficient DAB photooxidation.	18
				DAB photooxidation restricted for small samples.	
FLIPPER (>36 kDa)	P	+	+	Not functional in cytosol, since HRP-based.	19
				Large size.	
Halo/SNAP/CLIP-tag + TMR ligand (33/20/20 kDa)	L	+	+	DAB photooxidation restricted for small samples	This study

^*^P, peroxidase-based oxidation of DAB; L, light-based photooxidation of DAB.

^**^+, application possible; − application not possible without modification.
