# Supplementary material for: Self-labelling enzymes as universal tags for fluorescence microscopy, super-resolution microscopy and electron microscopy
Source: Sci Rep. 2015 Dec 8;5:17740. doi: 10.1038/srep17740 (PMC4672345; doi:10.1038/srep17740)
Supplement: Supplementary Information [file srep17740-s1.pdf]

Supplementary Materials

Self-labelling enzymes as universal tags for fluorescence microscopy, super-resolution microscopy and electron microscopy

Viktoria Liss, Britta Barlag, Monika Nietschke and Michael Hensel
Abt. Mikrobiologie, Universität Osnabrück, Osnabrück, Germany

Supplementary Material and Methods

Generation of transfection vectors

Plasmid pmTurquoise2-Golgi was digested with BsrGI and AgeI and the larger fragment was recovered. The insert of pSEMS-HaloTag-meGFP was recovered after digestion with BsrGI and AgeI and the fragments were ligated to generate p3986 with a Golgi targeting sequence (GTS). Plasmid pmPalmitoyl-mTurquoise2 was modified in the same way to generate p3988 with a palmitoylation signal for targeting to the inner leaflet of the cytoplasm membrane. SNAP-tag was amplified using primers SnapTag-For-BamHI + SnapTag-Rev-XbaI. The product was digested by BamHI/XbaI and replaced eGFP in p3432 to generate p3782.

The DNA fragment L16-CLIP-tag was synthesized by IDT (Coralville, USA), cloned in blunt end vector pJET1.2 and sequence confirmed to yield p4191. Using vectors with fusions of organelle markers to HaloTag, the HaloTag sequence was replaced against sequences encoding SNAP-tag or CLIP-tag using Gibson Assembly. Briefly p3986 and p3988 were amplified using the Vf-pSems and Vf-pSems. SNAP-tag and CLIP-tag were amplified using 1f-SNAP-pSems and 1r-SNAP-pSems. Combination of resulting vector and insert products are joined by an isothermal assembly reaction.

Using plasmid p3432, LAMP1 was amplified using hLAMP1-EcoRV-For and hLAMP1-AgeI-Rev. The resulting fragment, as well as plasmid pSems-LifeAct-HaloTag-meGFP were digested with AgeI and EcoRV and ligated to generate p3991. SNAP-tag and CLIP-tag were amplified using 1f-SNAP-pSems and 1r-SNAP-pSems and p3782 and p4191 as templates respectively. Resulting fragments as well as p3991 were digested with EcoRI and SbfI and ligated to generate p4323 and p4324.

Suppl. Tables

Table S 1. **Plasmids used in this study**

Designation	Genotype	Reference/source
pJET1.2	PCR fragment cloning vector	Fermentas/Thermo
p3432	LAMP1-eGFP	[1]
p3782	LAMP1-SNAP-tag	this study
p3986	Golgi-HaloTag-meGFP	this study
p3988	Palmitoyl-HaloTag-meGFP	this study
p3991	LAMP1-HaloTag-meGFP	this study
p4191	pJET1.2 L16-CLIP-tag	this study
p4296	Golgi-SNAP-tag-meGFP	this study
p4297	Golgi-CLIP-tag-meGFP	this study
p4298	Palmitoyl-SNAP-tag-meGFP	this study
p4299	Palmitoyl-CLIP-tag-meGFP	this study
p4323	LAMP1-SNAP-tag-meGFP	this study
p4324	LAMP1-CLIP-tag-meGFP	this study
pSems-LifeAct-HaloTag-meGFP		[2], Addgene
Palmitoyl-mTurquoise2		[3], Addgene
Golgi-mTurquoise2		[3], Addgene
Mito-HaloTag		[4], K. Busch
ER-FRP-HaloTag		[5]¸ J. Holthuis

Table S 2. Oligonucleotides and synthetic DNA used in this study

Designation	Sequence (5’-3’)
hLAMP1-EcoRV-For	tatgatatcATGGCGGCCCCCGGCAG
hLAMP1-AgeI-Rev	GCGACCGGTGGAtcccgg
SnapTag-For-BamHI	TATGGATCCGGCAGACAAAGACTGCGAAATG
SnapTag-Rev-XbaI	TTATCTAGATCAACCCAGCCCAGGCTTGCC
1f-SNAP-pSems	GTCGCCACCGAATTCCATATGGACAAAGACTGCGAAATGA AGCG
1r-SNAP-pSems	CGCGCCTATACCTGCAGGACCCAGCCCAGGCTTGCC
Vf-pSems	CCTGCAGGTATAGGCGCGCCAGGA
Vr-pSems	ATGGAATTCGGTGGCGACCGGTGGA

L16-CLIP-tag

GGCTCTGCGGCGTCTGCGGCGGGCGCGGGCGAAGCGGCGGCGATGGACAAAGACTGCGA
AATGAAGCGCACCACCCTGGATAGCCCTCTGGGCAAGCTGGAACTGTCTGGGTGCGAAC
AGGGCCTGCACCGTATCATCTTCCTGGGCAAAGGAACATCTGCCGCCGACGCCGTGGAAG
TGCCTGCCCCAGCCGCCGTGCTGGGCGGACCAGAGCCACTGATCCAGGCCACCGCCTGGC
TCAACGCCTACTTTCACCAGCCTGAGGCCATCGAGGAGTTCCCTGTGCCAGCCCTGCACC
ACCCAGTGTTCCAGCAGGAGAGCTTTACCCGCCAGGTGCTGTGGAAACTGCTGAAAGTGG
TGAAGTTCGGAGAGGTCATCAGCGAGAGCCACCTGGCCGCCCTGGTGGGCAATCCCGCC
GCCACCGCCGCCGTGAACACCGCCCTGGACGGAAATCCCGTGCCCATTCTGATCCCCTGC
CACCGGGTGGTGCAGGGCGACAGCGACGTGGGGCCCTACCTGGGCGGGCTCGCCGTGAA
AGAGTGGCTGCTGGCCCACGAGGGCCACAGACTGGGCAAGCCTGGGCTGGGTTACCCAT
ACGACGTCCCAGACTACGCTTAA

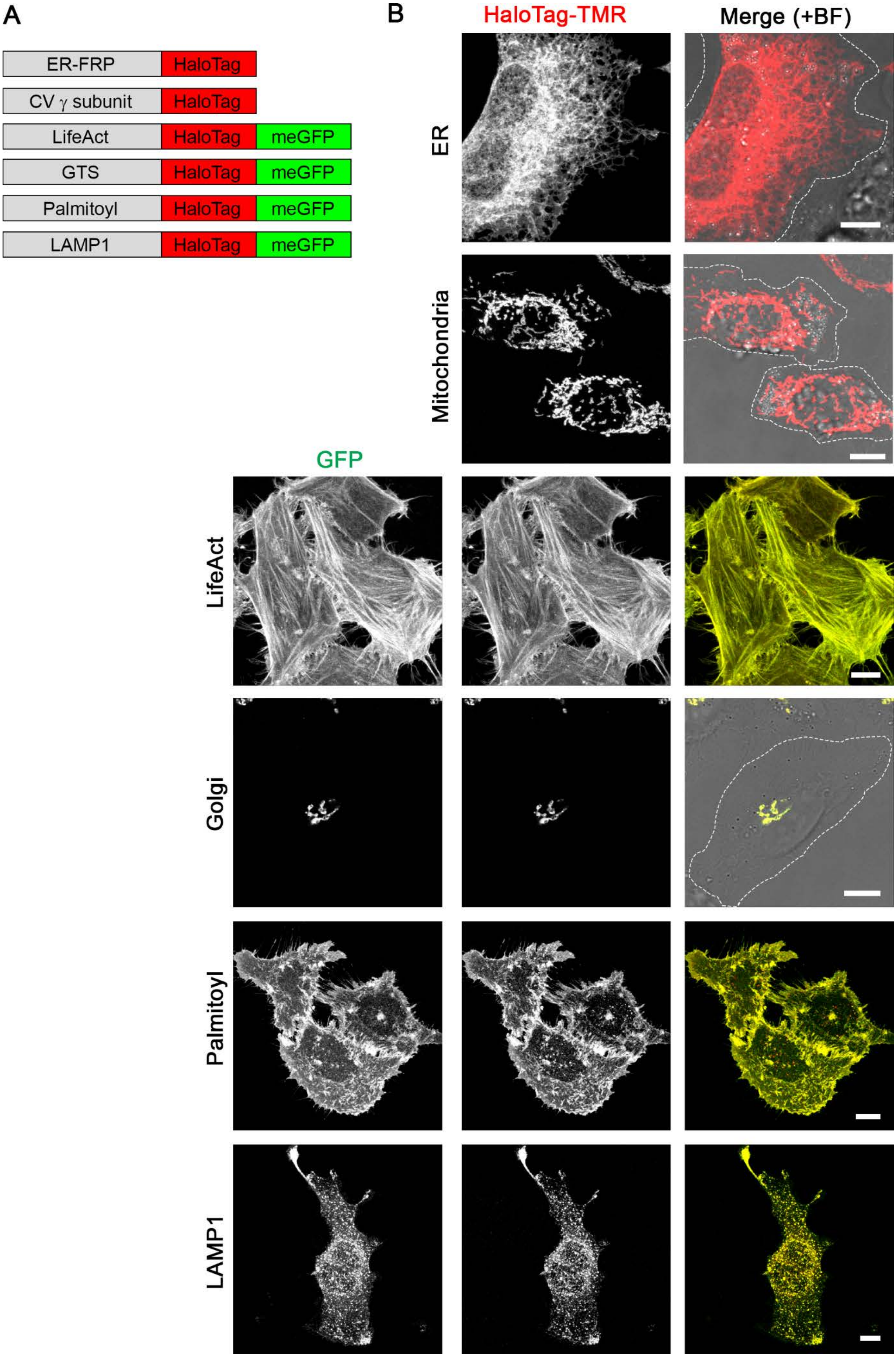
A
ER-FRP
HaloTag
CV γ subunit
HaloTag
LifeAct
HaloTag
meGFP
GTS
HaloTag
meGFP
Palmitoyl
HaloTag
meGFP
LAMP1
HaloTag
meGFP
B
HaloTag-TMR
Merge (+BF)
ER
Mitochondria
GFP
LifeAct
Golgi
Palmitoyl
LAMP1

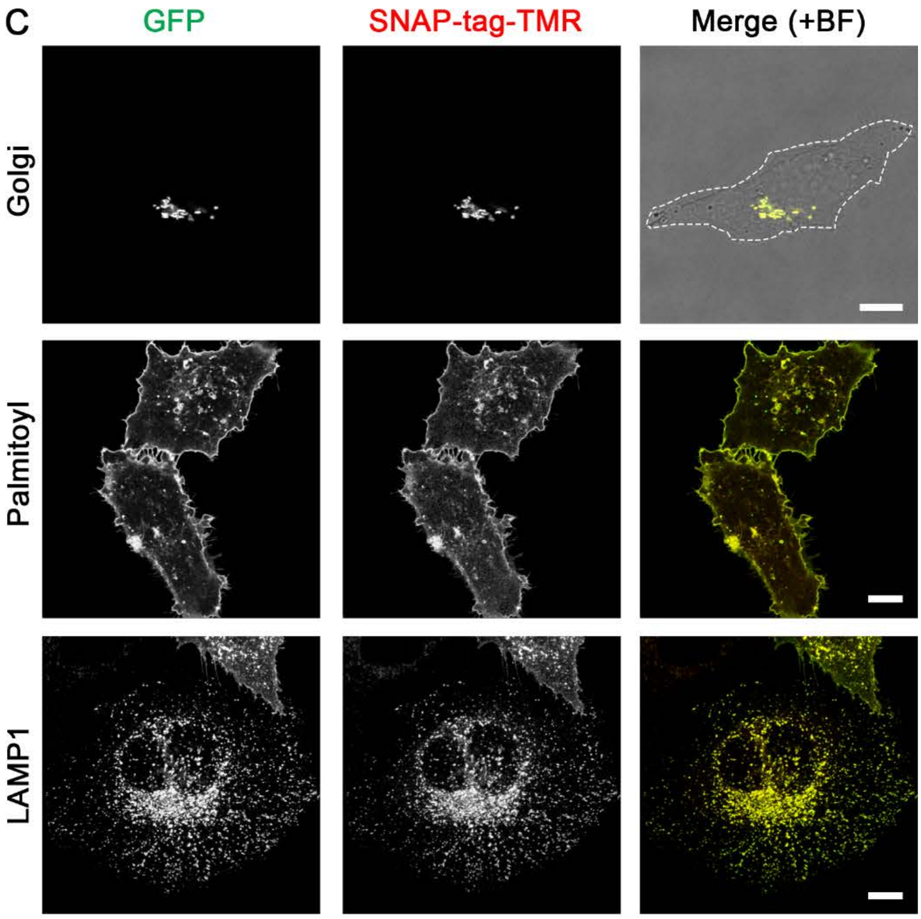
C
GFP
SNAP-tag-TMR
Merge (+BF)
Golgi
Palmitoyl
LAMP1

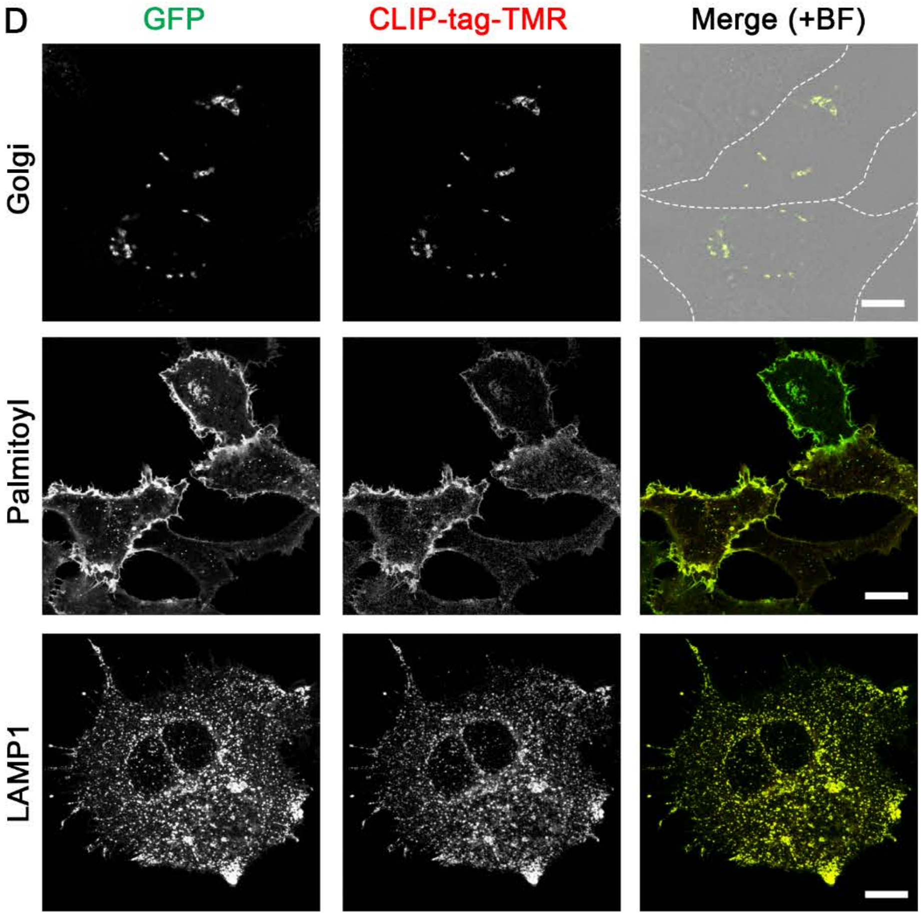
D
GFP
CLIP-tag-TMR
Merge (+BF)
Golgi
Palmitoyl
LAMP1

Fig. S1. **Self-labeling enzyme tags are suitable for various organelle marker proteins.** A) Overview of fusion proteins consisting of various organelle markers and HaloTag. Some fusion proteins also contained meGFP. The design of fusion proteins with SNAP-tag or CLIP-tag is identical, replacing HaloTag by either SNAP-tag or CLIP-tag. B) HeLa cells stably expressing LifeAct-HaloTag-meGFP [6] or transiently transfected with various plasmids encoding the indicated organelle markers fused to HaloTag were incubated with ligand HTL-TMR (red) for 15 min. to label the HaloTag followed by live cell imaging using CLSM. C) HeLa expressing SNAP-tag fusion of organelle marker. Staining was performed as for HaloTag. D) HeLa expressing CLIP-tag fusion of organelle marker. Staining was performed as for HaloTag. Cells were checked for expression, correct localization of organelle marker and unspecific background staining of TMR ligand by investigating the co-localization with GFP signal of some organelle markers. Scale bars: 10 µm.

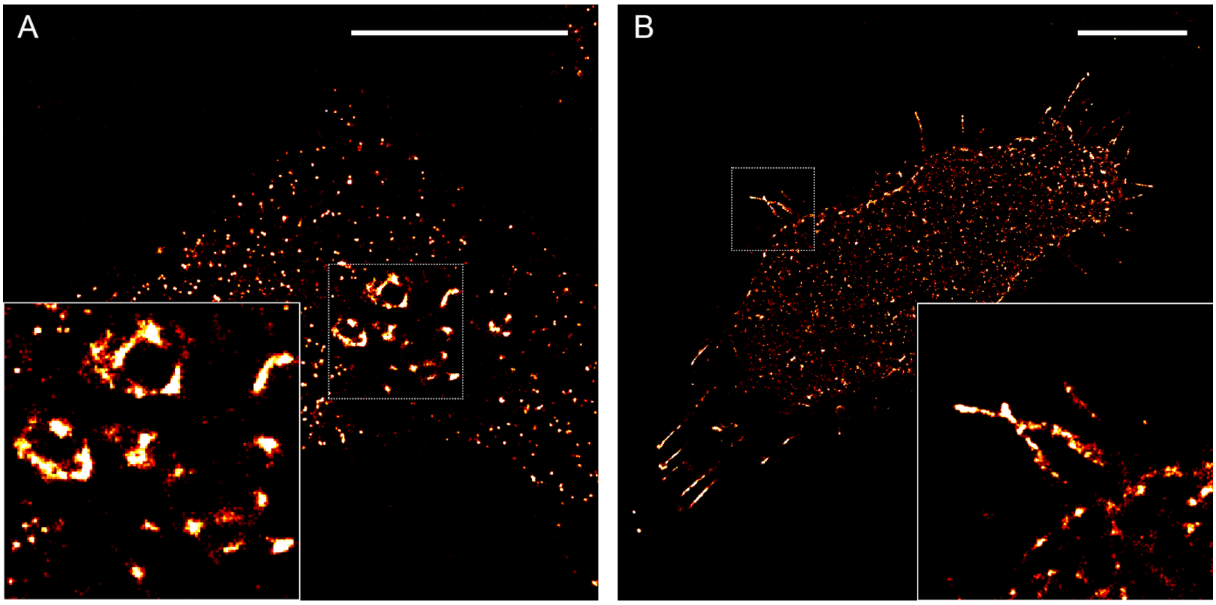

Fig. S2. **Super-resolution microscopy of organelle markers fused to HaloTag.** Cells were transfected for synthesis of Golgi-HaloTag-meGFP (A) or Palmitoyl-HaloTag-meGFP (B) and stained with 20 nM HTL-TMR for 45 min. After washing, cells were fixed with 3 % PFA and subjected to SRM. 1,000 frames with 32 ms exposure time were acquired using excitation with a 561 nm laser. Images were taken at time points of steady state of fluorescence intensity, i.e. when blinking TMR molecules were observed. Image series were localized using a modulated MTT version [2,7]. Scale bars: 10 µm.

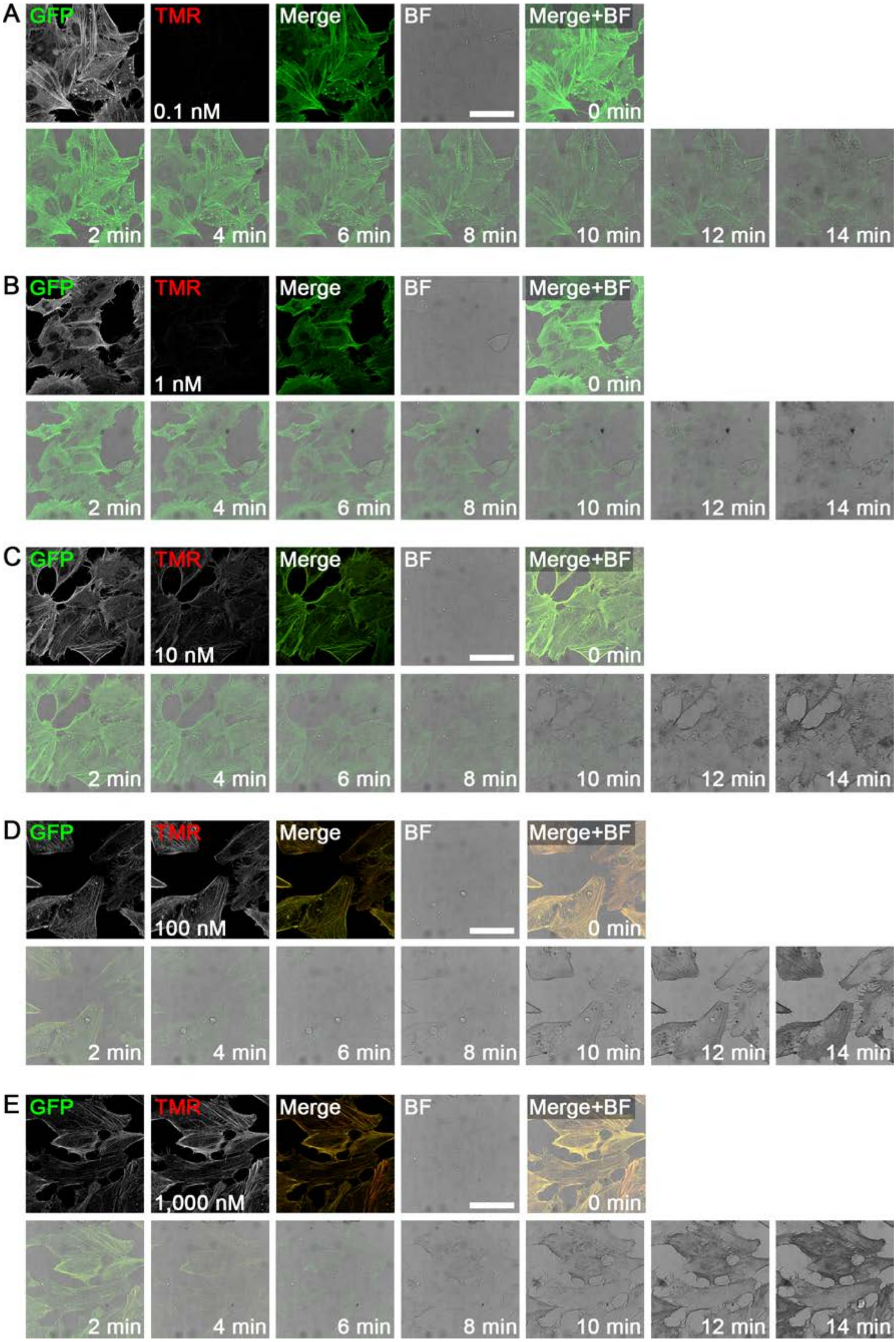

Fig. S3. **Time-dependent DAB photooxidation in HeLa cells expressing LifeAct-HaloTag-meGFP after labelling with various concentrations of HaloTag ligand coupled to TMR.** HeLa cells stably expressing LifeAct-HaloTag-meGFP were incubated for 15 min. with

medium containing HaloTag ligand coupled to TMR at concentrations ranging from 0.1 nM to 1,000 nM TMR. After fixation and quenching, cells were covered with fresh DAB solution in buffer and illuminated under the microscope for 14 min. with green light. Sufficient DAB photooxidation by TMR was observed for samples labelled with more than 10 nM TMR. Thereby a sufficient amount of DAB precipitates was formed after 12 min. with 10 nM TMR ligand and after 8-10 min. with 100 or 1,000 nM TMR ligand, with no significant difference between 100 nM and 1,000 nM. DAB photooxidation and DAB precipitate formation was concomitant with disappearance of fluorescence. Scale bars: 50 µm.

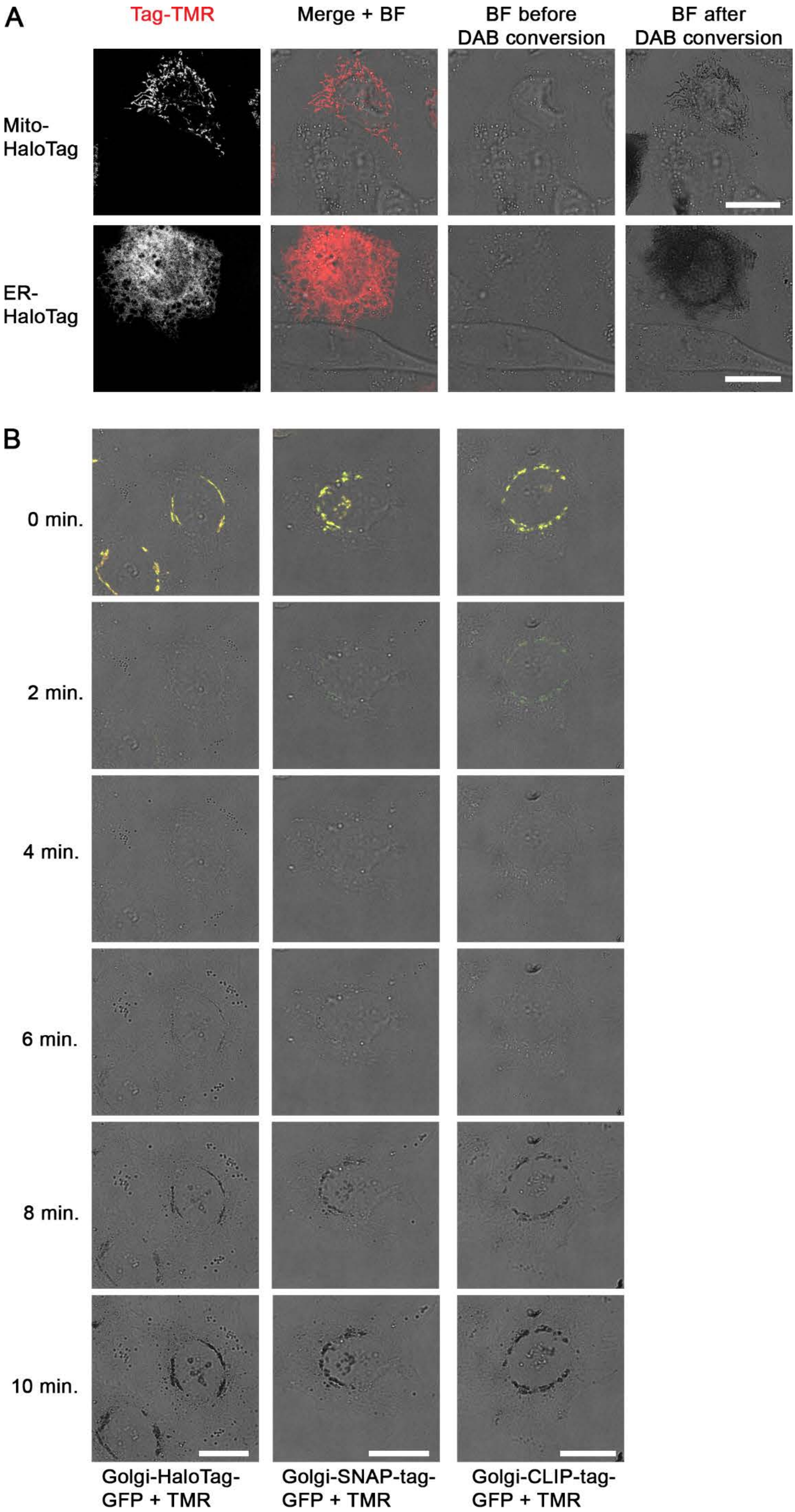
A
Tag-TMR
Merge + BF
BF before DAB conversion
BF after DAB conversion
Mito-HaloTag
ER-HaloTag
B
0 min.
2 min.
4 min.
6 min.
8 min.
10 min.
Golgi-HaloTag-GFP + TMR
Golgi-SNAP-tag-GFP + TMR
Golgi-CLIP-tag-GFP + TMR

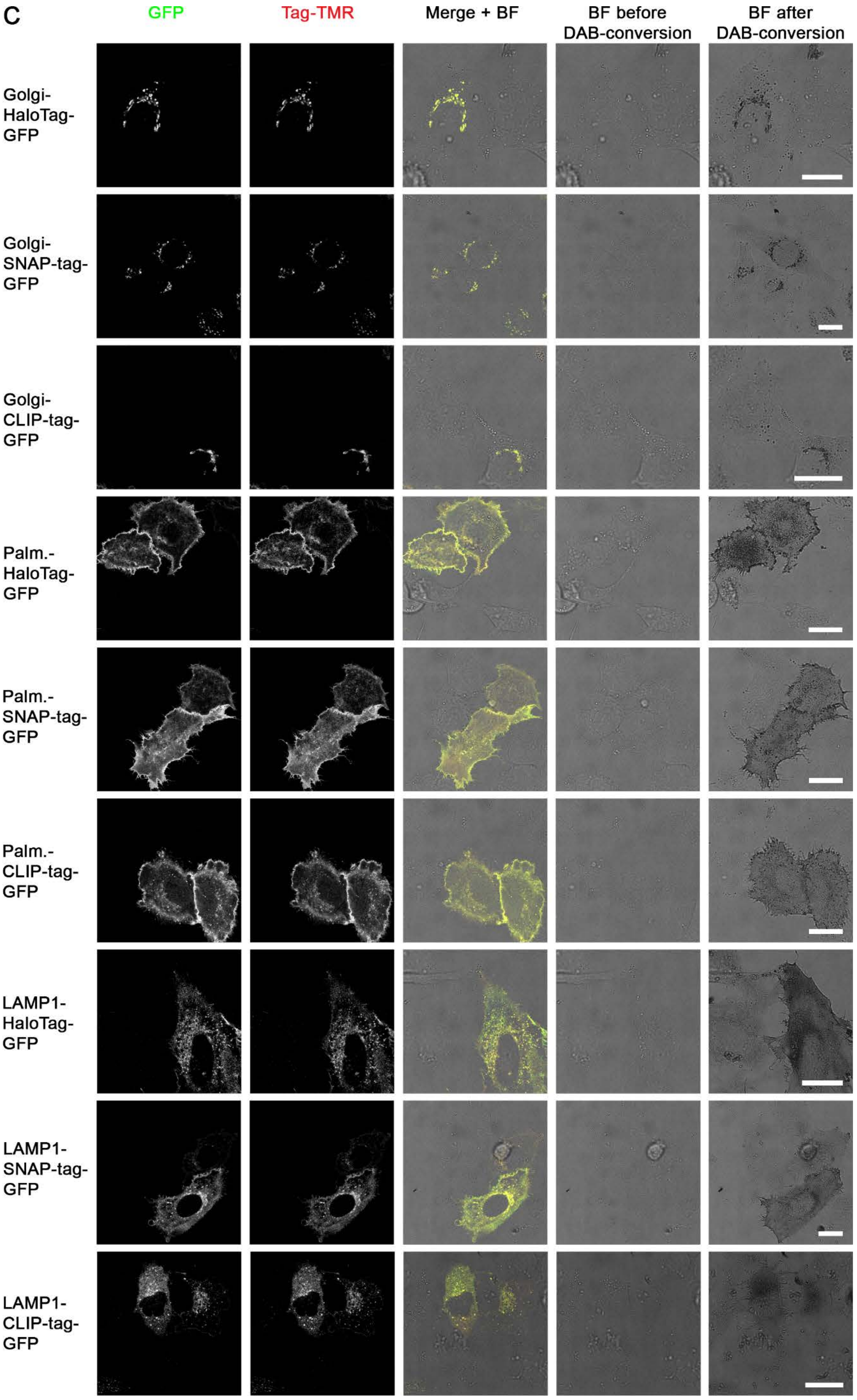
C
GFP
Tag-TMR
Merge + BF
BF before DAB-conversion
BF after DAB-conversion
Golgi-HaloTag-GFP
Golgi-SNAP-tag-GFP
Golgi-CLIP-tag-GFP
Palm.-HaloTag-GFP
Palm.-SNAP-tag-GFP
Palm.-CLIP-tag-GFP
LAMP1-HaloTag-GFP
LAMP1-SNAP-tag-GFP
LAMP1-CLIP-tag-GFP

Fig. S4. **DAB photooxidation in HeLa cells expressing various organelle marker fused to HaloTag, SNAP-tag or CLIP-tag after labelling with TMR ligands.** HeLa cells expressing various organelle markers fused to HaloTag, SNAP-tag or CLIP-tag as indicated were incubated with medium containing 100 nM of specific ligands coupled to TMR for 15 min. After fixation and quenching, cells were covered with fresh DAB solution in buffer. During illumination of samples under the microscope, samples labelled with TMR and incubated with DAB solution showed the formation of localized DAB polymer after 8-12 min., concomitant with disappearance of fluorescence. A) Fluorescence and DAB polymer formation by photooxidation through various TMR-labelled organelle markers containing GFP-tags. B) Fluorescence and DAB polymer formation by photooxidation through TMR-labelled fusion proteins without GFP-tag. C) Time dependence of DAB polymer formation by photooxidation in cells expressing GTS fused to HaloTag, SNAP-tag or CLIP-tag. Note the absence of unspecific DAB polymer formation in non-transfected cells. Scale bars: 20 µm.

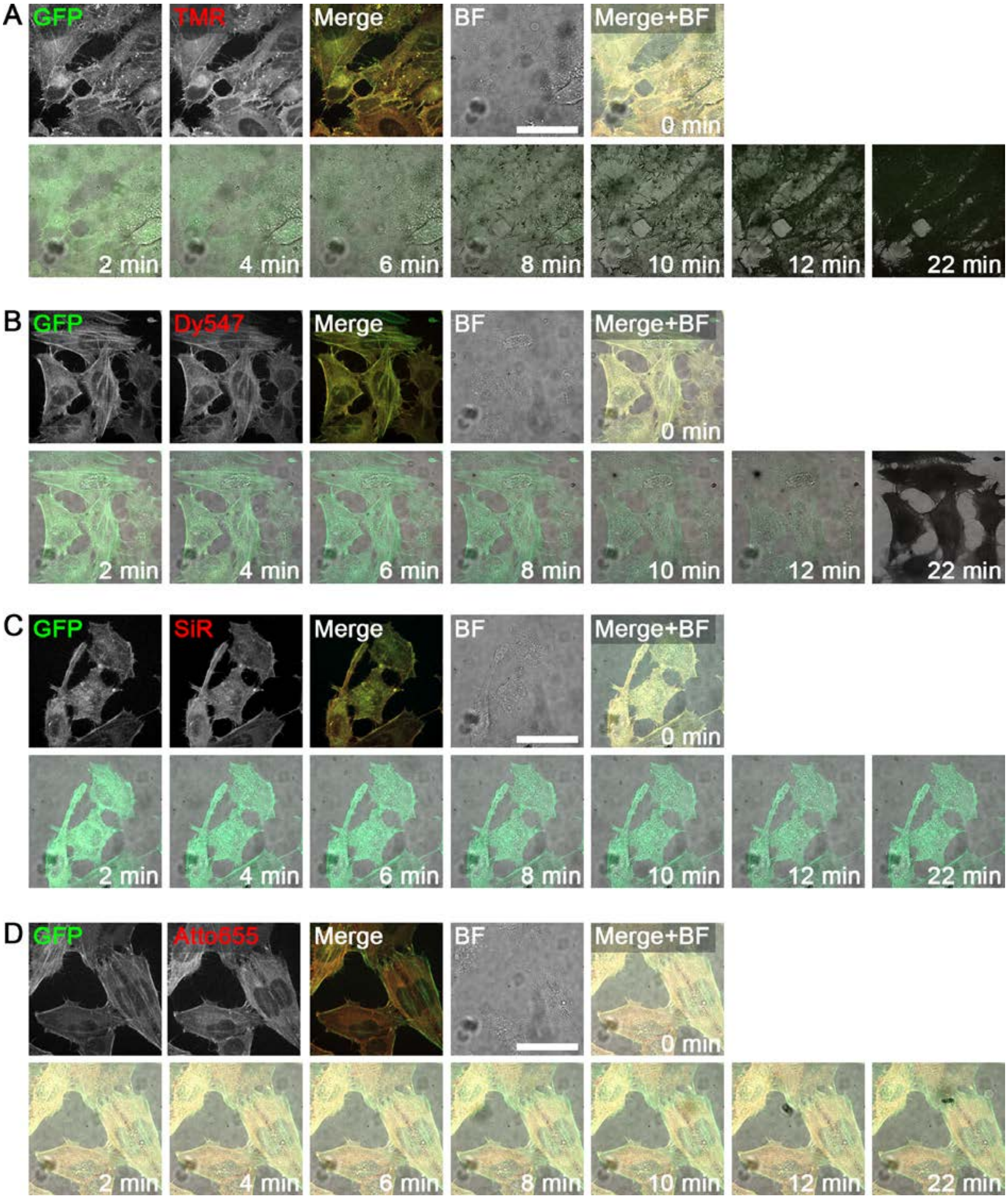

Fig. S5. **DAB photooxidation in HeLa cells expressing LifeAct-HaloTag-meGFP after labelling with HaloTag ligand coupled to various fluorochromes.** HeLa cells stably expressing LifeAct-HaloTag-meGFP were incubated with medium containing 100 nM of HaloTag ligand coupled to the indicated fluorochromes for 22 min. After fixation and quenching, cells were covered with fresh DAB solution in buffer and illuminated under the microscope at the fluorochrome-specific excitation light. A) TMR and B) Dy547 were illuminated with green light (537-563 nm) and C) SiR and D) Atto655 with red light (625-655

nm). Samples labelled with TMR ligand showed the formation of localized DAB polymer after 8 min. (A) and samples labelled with Dy547 ligand after 16 min. (B), in both cases concomitant with disappearance of fluorescence. SiR ligand (C) and Atto655 ligand (D) led to no DAB photooxidation within 22 min. Scale bars: 50 µm.

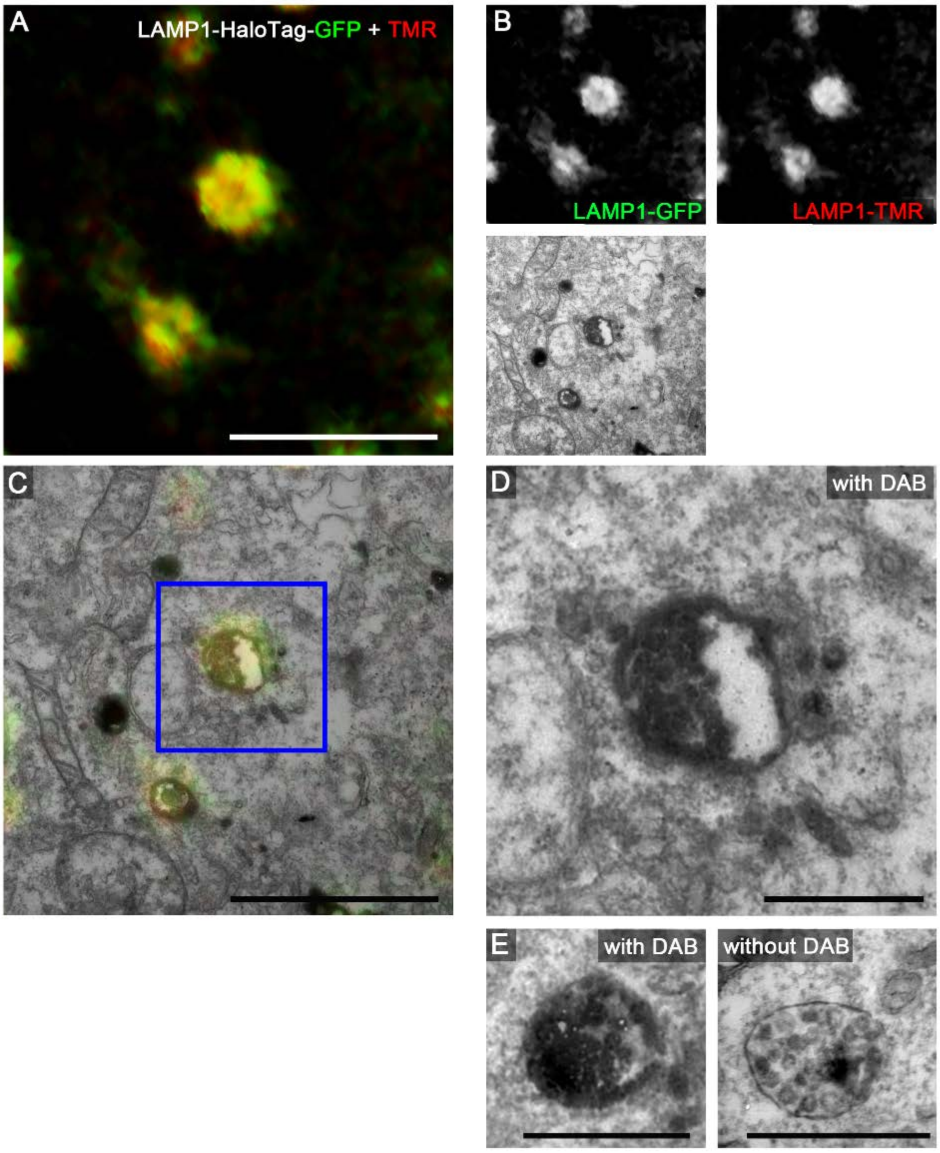

Fig. S5. **HaloTag-TMR as genetically encoded CLEM marker for proteins in late endosomes.** HeLa cells transiently transfected with LAMP1-HaloTag-meGFP were seeded on a Petri dish with a gridded coverslip and treated as described for Fig. 3. Scale bars: 2 µm (A, C), 500 nm (D, E).

References

1 Rajashekar, R., Liebl, D., Seitz, A. & Hensel, M. Dynamic remodeling of the endosomal system during formation of *Salmonella*-induced filaments by intracellular *Salmonella enterica*. *Traffic* **9**, 2100-2116, doi:TRA821 [pii] 10.1111/j.1600-0854.2008.00821.x (2008).

2 Wilmes, S. *et al.* Triple-color super-resolution imaging of live cells: resolving submicroscopic receptor organization in the plasma membrane. *Angew Chem Int Ed Engl* **51**, 4868-4871, doi:10.1002/anie.201200853 (2012).

3 Goedhart, J. *et al.* Structure-guided evolution of cyan fluorescent proteins towards a quantum yield of 93%. *Nat Commun* **3**, 751, doi:10.1038/ncomms1738 (2012).

4 Wilkens, V., Kohl, W. & Busch, K. Restricted diffusion of OXPHOS complexes in dynamic mitochondria delays their exchange between cristae and engenders a transitory mosaic distribution. *J Cell Sci* **126**, 103-116, doi:10.1242/jcs.108852 (2013).

5 Csordas, G. *et al.* Imaging interorganelle contacts and local calcium dynamics at the ER-mitochondrial interface. *Mol Cell* **39**, 121-132, doi:10.1016/j.molcel.2010.06.029 (2010).

6 Deerinck, T. J. *et al.* Fluorescence photooxidation with eosin: a method for high resolution immunolocalization and in situ hybridization detection for light and electron microscopy. *J Cell Biol* **126**, 901-910 (1994).

7 Serge, A., Bertaux, N., Rigneault, H. & Marguet, D. Dynamic multiple-target tracing to probe spatiotemporal cartography of cell membranes. *Nat Methods* **5**, 687-694, doi:10.1038/nmeth.1233 (2008).
